# Inclusion body myositis with early onset: a population-based study

**DOI:** 10.1007/s00415-023-11878-w

**Published:** 2023-07-27

**Authors:** Ulrika Lindgren, Carola Hedberg-Oldfors, Rille Pullerits, Christopher Lindberg, Anders Oldfors

**Affiliations:** 1 Department of Laboratory Medicine, Institute of Biomedicine, Sahlgrenska Academy, University of Gothenburg, Gothenburg, Sweden; 2 Neuromuscular Center, Department of Neurology, Sahlgrenska University Hospital, Gothenburg, Sweden; 3 Department of Rheumatology and Inflammation Research, Institute of Medicine, Sahlgrenska Academy, University of Gothenburg, Gothenburg, Sweden; 4 Department of Clinical Immunology and Transfusion Medicine, Sahlgrenska University Hospital, Gothenburg, Sweden

**Keywords:** Inclusion body myositis, Inflammatory myopathy, Muscular weakness, Mitochondrial DNA, Epidemiology

## Abstract

**Introduction:**

Inclusion body myositis (IBM), an inflammatory myopathy with progressive weakness without efficient treatment, typically presents after 45 years of age and younger patients are sparsely studied.

**Methods:**

In a population-based study during a 33-year period, 142 patients with IBM were identified in western Sweden. Six patients fell outside the European Neuromuscular Centre 2011 criteria for IBM due to young age at symptom onset, verified by a muscle biopsy < 50 years of age. These were defined as early-onset IBM and included in this study. Medical records, muscle strength, comorbidities, muscle biopsies, and nuclear- and mitochondrial DNA were examined and compared with patients with IBM and age matched controls from the same population.

**Results:**

The median age at symptom onset was 36 (range 34–45) years and at diagnosis 43 (range 38–58) years. Four patients were deceased at a median age of 59 (range 50–75) years. The median survival from diagnosis was 14 (range 10–18) years. The prevalence December 31 2017 was 1.2 per million inhabitants and the mean incidence 0.12 patients per million inhabitants and year. The mean decline in quadriceps strength ± 1 standard deviation was 1.21 ± 0.2 Newton or 0.91 ± 0.2% per month and correlated to time from diagnosis (p < 0.001). Five patients had swallowing difficulties. All patients displayed mitochondrial changes in muscle including cytochrome c oxidase deficiency and the mitochondrial DNA mutation load was high.

**Conclusions:**

Early-onset IBM is a severe disease, causing progressive muscle weakness, high muscle mitochondrial DNA mutation load and a reduced cumulative survival in young and middle-aged individuals.

## Introduction

The inflammatory myopathy inclusion body myositis (IBM) typically presents with progressive muscle weakness and atrophy affecting the quadriceps muscle and finger flexors, or swallowing difficulties. Most affected individuals are middle-aged or older. The gold standard for diagnosis is clinical evaluation combined with muscle biopsy, where histopathological findings include inflammation, rimmed vacuoles, protein aggregates and signs of mitochondrial pathology with cytochrome c oxidase (COX) deficient muscle fibers.

The commonly applied European Neuromuscular Centre (ENMC) 2011 criteria for IBM requires symptom onset > 45 years of age for diagnosis [1]. Although an age criterion is important to avoid inclusion of other myopathies with similar pathology in studies on IBM cohorts, it may also cause exclusion of true patients with IBM. Studies on unusually early-onset IBM may give important clues to pathogenesis and provide information about disease progression without confounding factors caused by old age. There are no population-based studies on young patients with IBM, and thus far they have only been reported in single case studies or as part of larger cohort studies [2–5].

In a recent study, we used a population-based design to identify all patients fulfilling the ENMC 2011 criteria for clinicopathological IBM in Region Västra Götaland (VGR), western Sweden, between January 1, 1985 and December 31, 2017, therefore excluding patients with symptom onset at 45 years of age and younger [1, 6]. This resulted in a population-based group of young patients fulfilling all criteria except age and gave us the rare opportunity to describe them in comparison to a well-defined and population matched cohort of patients with clinicopathological IBM.

In this study, we aimed to describe epidemiology, survival, clinical parameters including progression of weakness, and mitochondrial pathology in patients with early-onset IBM, defined as fulfillment of all ENMC 2011 criteria for clinicopathological IBM except the age criterion, in a population-based retrospective case series. Clinicopathological IBM was defined as fulfillment of all ENMC 2011 research diagnostic criteria for clinicopathological IBM, and the term IBM was used to describe inclusion body myositis in general.

## Materials and methods

### Workflow and population data

Patients were identified from the regional referral centers for muscle biopsy diagnostics and neuromuscular disorders at the Department of Pathology and the Department of Neurology, both at Sahlgrenska University Hospital, Gothenburg, Sweden as previously described [6]. Archival muscle biopsy specimens were reevaluated in all patients with suspected IBM. The date of diagnosis was defined as the date of the first biopsy needed to fulfill the pathological features of ENMC 2011 research diagnostic criteria for clinicopathological IBM [1].

All available medical records and diagnosis codes for the included patients were manually reviewed up to June 30, 2018. Laboratory data were gathered from the first available visit after symptom onset. Muscle weakness distribution for diagnosis and myometer values for quadriceps, both performed by a few experienced investigators, were also collected. Autoimmune diseases were defined as the 30 diseases listed in Eaton et al. (excluding myositis), including amongst others Sjogren’s syndrome and psoriasis [7]. Malignancies were defined according to the NORDCAN database [8, 9].

### Inclusion and exclusion criteria

Inclusion criteria were (a) fulfillment of ENMC 2011 research diagnostic criteria for clinicopathological IBM except the age criteria [1]; (b) diagnostic muscle biopsy from the period January 1, 1985, to December 31, 2017; (c) inhabitant in VGR at any time during the studied period; (d) symptom onset < 46 years of age and (e) first muscle biopsy with endomysial inflammatory cell infiltrates < 50 years of age.

Exclusion criteria were (a) probability of another or additional muscle disease based on all available information evaluated by an expert group (authors UL, CH-O, CL and AO).

Individuals fulfilling these criteria were considered patients with early-onset IBM in this study.

A limit of < 50 years of age at the first muscle biopsy with inflammation was used to verify probable onset of symptoms caused by IBM before 46 years of age.

### Muscle biopsies and serum samples

Muscle biopsies and evaluation of morphology were performed as earlier described [6]. Immunohistochemical staining of p62/ sequestosome-1 was used as a marker for protein aggregates in vacuolated muscle fibers.

Analysis of serum samples and autoantibodies were performed in all patients with early-onset IBM with available material, and each patient was paired with three age- and sex matched blood donors from VGR. The analysis included a line blot with 16 autoantibodies associated with autoimmune inflammatory myopathies and separate analysis of antibodies against cytosolic 5′-nucleotidase 1A (cN1A) and 3-hydroxy-3-methylglutaryl coenzyme A (HMGCR) [6].

### Whole genome sequencing

Total genomic DNA was extracted from the muscle biopsy specimens using standard protocols. Whole genome sequencing (WGS) was performed using the TruSeq™ PCR free library preparation kit (Illumina, San Diego, CA, USA) and sequenced on the Illumina’s HiSeq X or the Illumina NovaSeq 6000 platforms (Illumina, San Diego, CA, USA). The paired-end reads from the WGS were aligned to the reference genome (hg19) and variant calling for single-nucleotide variants (SNVs) and small indels in nuclear and mitochondrial DNA was performed using Sentieon DNAscope. The variants were further filtered for identification of potentially pathogenic variants in candidate genes associated with myopathy using an in-silico gene panel based on The GeneTable of Neuromuscular Disorders 2022 (www.musclegenetable.fr/) and variants in the mitochondrial DNA (mtDNA). Genes and variants were further manually curated based on literature search, the clinical information and muscle biopsy findings.

Disease controls consisted of 16 patients with clinicopathological IBM, aged 51–75 years at biopsy. Normal controls consisted of two groups of age- and sex matched individuals with normal muscle biopsies. Nine individuals aged 41–60 years served as normal controls for early-onset IBM, and eight individuals aged 61–80 years for clinicopathological IBM.

Large mtDNA deletions and duplications were analyzed using the WGS data by an in-house bioinformatic tool called MitoSAlt as previously described, except that mean coverage depth was based on the entire mtDNA [10, 11].

Copy number of mtDNA in relation to nuclear DNA was estimated as previously described: mtDNA copy number = mitochondrial genome coverage × 2/nuclear genome coverage [10, 12].

### Statistical analyses

For survival calculations, 128 patients (89 men and 39 women) in the same population with clinicopathological IBM as defined by ENMC 2011 served as disease control group [1, 6]. All inhabitants in VGR matched for age, year and sex for patients with early-onset IBM and clinicopathological IBM respectively served as control groups, using data from Statistics Sweden [13]. For calculations of prevalence and incidence, the population in VGR was used as control group, using data from Statistics Sweden [13]. Epidemiological data (cumulative survival, prevalence and incidence) were adjusted for one patient relocating during the studied time period. Spearman’s rank correlation coefficient was used for analysis of muscle weakness, and group comparisons of mitochondrial DNA data were performed with the Mann–Whitney U test. For all analyses, p < 0.05 was considered significant, with adjustments for multiple analyzes when applicable.

Calculations were performed using Microsoft Excel for Mac 2011 (Microsoft Corporation, Redmond, WA, USA) and Graph Pad Prism 9.4.1 for MacOS (GraphPad Software, San Diego, CA, USA).

## Results

Six patients with possible IBM, symptom onset < 46 years of age and a muscle biopsy showing endomysial inflammatory cell infiltration < 50 years of age were identified during the studied time period. All six patients fulfilled the ENMC 2011 criteria for clinicopathological IBM except the age criteria and were included in the study. No patients were excluded due to probability of another muscle disease. Three patients with early-onset IBM were men and three women. Median age at diagnosis was 43 years and the median age at symptom onset was 36 years, resulting in a median diagnostic delay of 6 years (Table 1). The median follow-up time from diagnosis to June 30, 2018, or death was 11 years.

**Table 1:** Patient characteristics

General data	Mean; median (range) (y)
Age at symptom onset	38; 36 (34–45)
Age at diagnosis	45; 43 (38–58)
Diagnostic delay	7; 6 (2–15)
Age at death, n = 4	61; 59 (50–75)
Follow up time from diagnosis (y)	12; 11 (5–18)
Years from symptom onset	
Dysphagia, n = 5	3; 3 (0–7)
Ventilation assistance, n = 3	20; 14 (13–32)
Wheelchair use, n = 5	14; 16 (8–19)

Presenting symptom	n
Quadriceps weakness	2
Quadriceps and neck flexion weakness	1
Finger flexor weakness	1
Dysphagia	1
Hip extension weakness	1

*y* years, *n* number of patients

### Survival, prevalence, and incidence

Four patients with early-onset IBM were deceased, at a median age of 59 years (Table 1). Median survival from diagnosis was 14 years (mean 14 years, range 10–18 years) and from symptom onset 19 years (mean 22 years, range 16–32 years). For the population in VGR, mean expected survival at 50 years of age during 1994–2017 were 30 years for men and 34 years for women [13], corresponding to 80 and 84 years of age at death respectively. Of the four deceased patients, the cause of death was reported as respiratory insufficiency in one patient, pneumonia in one patient, and data were not available for two patients.

**Fig. 1:**
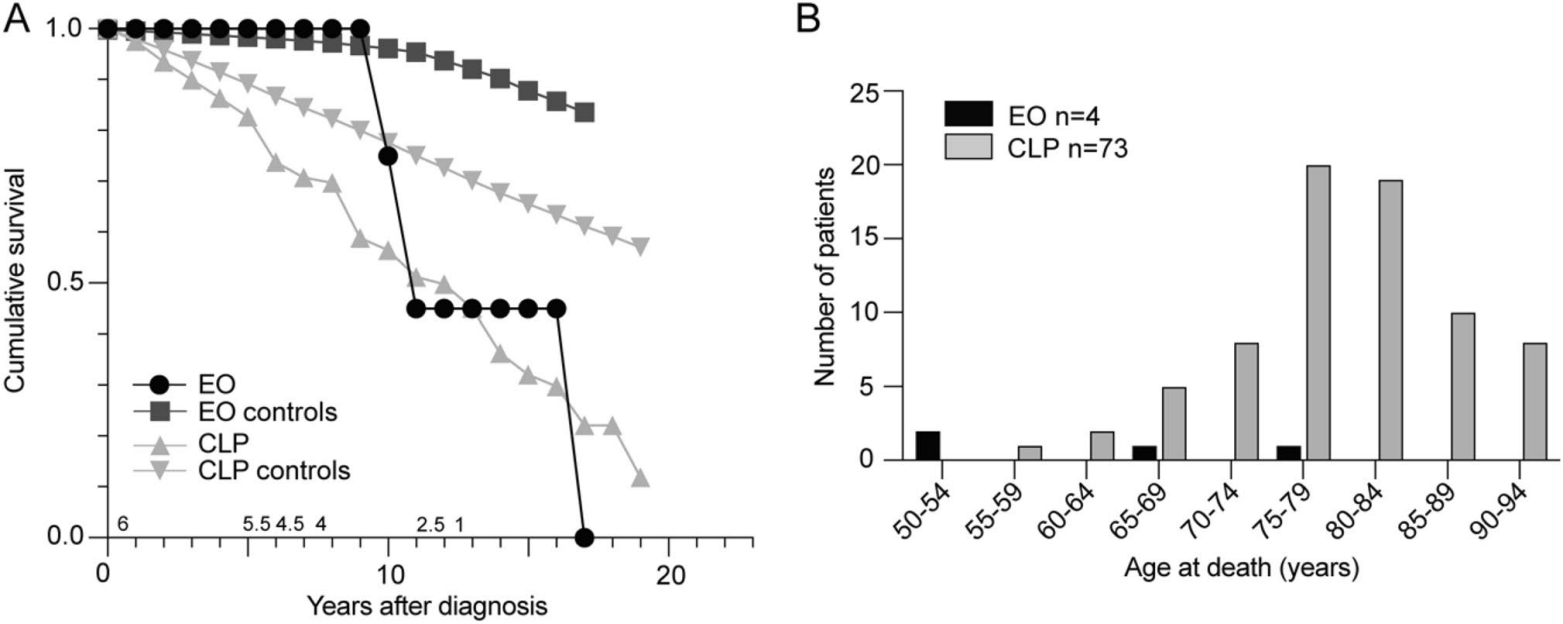
Survival in IBM. **A** Life table estimate of observed cumulative survival in early-onset IBM, clinicopathological IBM, and matched controls. The number above the x-axis shows the mean number of patients with early-onset IBM at risk per year. The number of controls for patients with early-onset IBM varied from 6081 to 11,406 depending on age and year. **B** Age at death in patients with early-onset IBM and clinicopathological IBM in VGR, Sweden. *EO* early-onset IBM, *CLP* clinicopathological IBM

Cumulative survival of the six patients with early-onset IBM was compared to the age- and sex- matched population and to 128 patients with clinicopathological IBM in the same population (Fig. 1A) [6]. From 10 years after diagnosis, patients with early-onset IBM had a decreasing cumulative survival compared both to the matched population and patients with clinicopathological IBM. Median age at death was 59 years in early-onset IBM and 80 years in clinicopathological IBM in the same population (Fig. 1B) [6]. Due to the low number of patients with early-onset IBM, no statistical analysis was performed.

December 31, 2017, VGR had 1.7 million inhabitants, and two of the patients with early-onset IBM were alive, resulting in a prevalence of 1.2 patients with early-onset IBM per million inhabitants. The mean incidence of early-onset IBM 1985 to 2017 was 0.12 per million inhabitants and year.

### Signs and symptoms

All patients had weakness in muscles typically involved in IBM, including finger flexor and quadriceps muscles. Neck flexion weakness was reported in three patients. Maximal serum CK levels were < 15 times upper limit of normal (ULN) in all patients. Electromyography (EMG) results were available for five patients, who all showed a myopathic pattern. Occasional myotonic discharges were found in the biceps brachii muscle in one patient, but were not present in other examined muscles or in repeated examination. Muscle magnetic resonance tomography (MRI) was performed in one patient and showed atrophy and fat tissue replacement in the distal part of quadriceps femoris muscle, and the anterior compartment of the lower leg. Electrocardiogram was assessed without cardiac conduction defects in all patients. Two patients had left ventricular hypertrophy, one presenting after an episode of Takutsubo cardiomyopathy during a severe pneumonia, and one after several years of hypertonia. In the remaining four patients, there were no records of cardiac involvement.

The most common first symptom was quadriceps weakness (Table 1). Less common muscle symptoms during the disease course included two patients reporting myalgia, and dysarthria, lagophthalmos, finger extension weakness, atrophy of the sternocleidomastoid muscle, atrophy of upper arm, atrophy of lower leg, shoulder atrophy and laryngospasms during sleep reported in one patient each.

Dysphagia was reported in five patients and two had undergone one or more surgical treatments for dysphagia.

Recurrent pneumonia was reported in three patients, all of whom died during the follow up time. Three patients had ventilation assistance, which was started 6, 8 and 15 years from diagnosis. One patient had tracheostomy and a bilevel positive airway pressure breathing device, one an unspecified nighttime ventilator and the third a cough machine. All three patients deceased within four years after start of ventilation assistance.

### Muscle strength

**Fig. 2:**
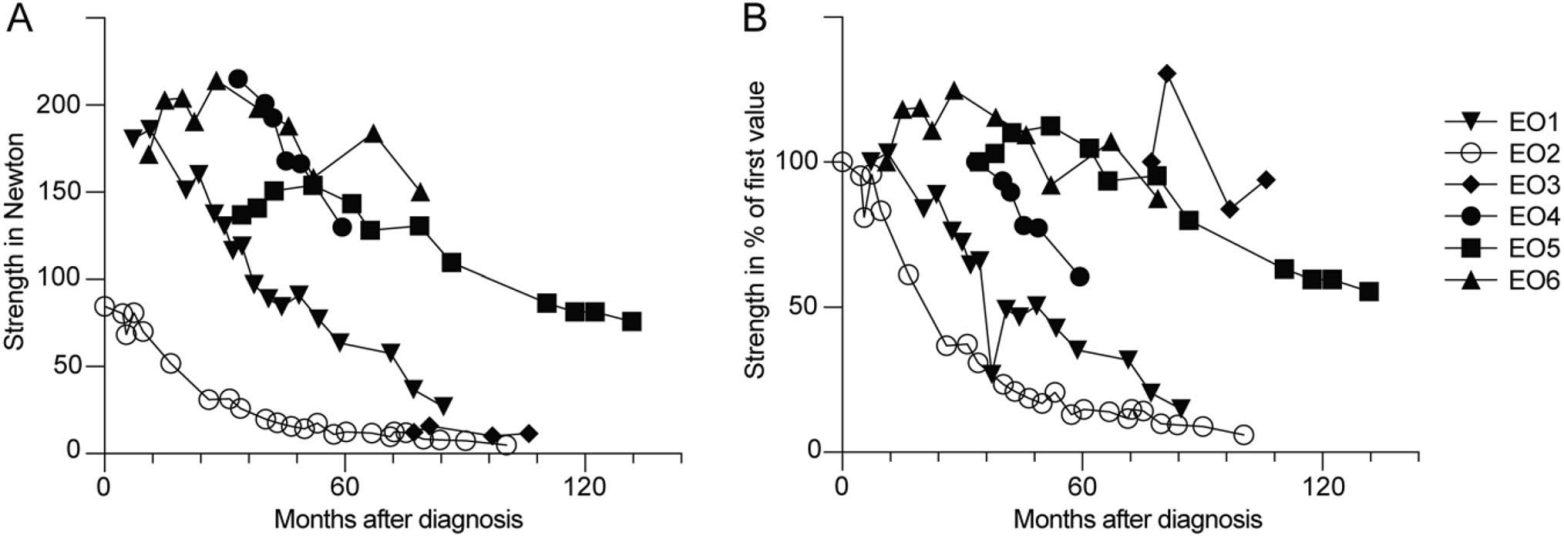
Development of knee extension weakness in individual patients over time from diagnosis. **A** Absolute strength in Newton (N) range ± 1SD − 0.11 ± 0.10 N to − 3.4 ± 0.31 N per month. **B** Strength as % of the first available measurement range ± 1SD − 0.32 ± 0.15% to − 1.6 ± 0.14% per month. *EO* early-onset IBM, *SD* standard deviation

Loss of muscle strength was evaluated as the development of knee extension weakness over time (Fig. 2). A hand-held dynamometer was used for measurements, and the mean value for the left and right sides combined was calculated. The mean ± 1 standard deviation (SD) decline was 1.21 ± 0.2 Newton or 0.91 ± 0.2% per month and correlated with time from diagnosis (p < 0.001).

### Comorbidities

Autoimmune disease as defined by Eaton et al. [7] was found in one patient with early-onset IBM, who had both Sjogren’s syndrome and psorisasis. Raynaud syndrome was reported in two other patients, and one had suspected systemic lupus erythematous (SLE). One patient had iritis, hypogammaglobulinemia and interstitial lung fibrosis.

Hepatitis C virus antibodies were not positive in any of the three patients tested, and HIV antibodies were negative in the four patients tested. Information on HTLV-1 was not available in any patient.

No malignancies were reported during the follow-up time except one patient with basal-cell carcinoma. For the control group consisting of the population in the Western Region, Sweden, the risk of malignancy before 75 years of age during 2012 to 2016 was 31% for men and 28% for women [8, 9].

### Immunomodulating treatment

All patients had received corticosteroids and immunomodulating treatment after symptom onset. One individual was treated with corticosteroids for 4 days, the remaining patients for 1–15 years. None of the patients had any history of using statins. Each patient had tried one to five different immunomodulating treatments. Three patients had more than one drug simultaneously. The most tried immunomodulating treatment was mycophenolate mofetil and methotrexate (n = 3), followed by abatacept, azathioprine, ciclosporin and intravenous immunoglobulin (n = 2) and least common were anakinra, anti-thymocyte globulin and interferon β-1a (n = 1).

The median treatment time with immunomodulatory therapy, excluding corticosteroids, was 60 months (range 14–197 months). One patient received immunoglobulin as treatment for hypogammaglobulinemia, resulting in an outlier value for time of treatment by start of treatment 178 months before diagnosis. Excluding this patient, the mean time from diagnosis to start of treatment was 23 months (range 4–49 months).

Four patients reported a transient improvement after treatment with corticosteroids and in one also after treatment with methotrexate. Another patient reported an initial increase of strength after starting methotrexate, but then deteriorated. A possibly slower disease course than expected was seen in one patient using the combination of mycophenolate mofetil and abatacept. Due to the small number of patients, no comparison of treatment response was possible between patients with and without autoimmune diseases. No clear differences were seen between the progression of knee extension weakness during periods with and without immunomodulating treatment (Fig. 2).

### Autoantibodies

Analysis of autoantibodies was performed in two patients and compared to six matched controls. The remaining four patients had no material available for analysis. Anti-cN1A and anti-Ro52 was positive in one patient respectively. One control was positive for anti-cN1A and one for Pl-12. Anti-HMGCR was not positive in any patient or control. Medical records reported positive antinuclear antibody (ANA) in two patients, negative ANA in three patients and negative anti-SSA in two patients.

### Biopsy data

All patients with early-onset IBM fulfilled the ENMC 2011 pathological criteria for diagnosis. Five of the six patients with early-onset IBM had two or more muscle biopsies, and a total of 19 muscle biopsies were assessed (Table 2). MHC-I was positive in a typical pattern for IBM in all patients and MHC-II in five patients with available material. Morphological and mtDNA findings varied over time and/or assessed muscle in some of the individuals (Table 2). Patient EO5 had three simultaneous biopsies from the tibialis anterior, quadriceps vastus lateralis and biceps muscles, which showed differences in the number of COX-deficient fibers, rimmed vacuoles and p62-positive inclusions as well as mutation load. The same quadriceps vastus lateralis muscle was biopsied twice during the same year, with the mtDNA mutation load varying between 1.2 and 6.2%. Some patients had only lesser differences between biopsies, including patient EO2 who had four subsequent biopsies from the deltoid muscle. There were no inflammatory cell infiltrates, fibrosis or atrophy in the control muscle biopsies, but occasional COX-deficient fibers occurred in some of them as expected.

**Table 2:** Muscle biopsy data in six patients with early-onset IBM

Patient	Muscle	Age at biopsy (years)	Inflammation	RV	p62	COX deficiency	Fibrosis	Copy number	Mutation load (%)
EO1	Vastus	41	++	++	++	3%	+	2174	2.85
EO2	nd^a^	38	+++	nd	nd	nd	+		
	Delt sin^a^	39	+++	++	++	8%	+		
	Delt sin^a^	39	+++	+++	+++	11%	+		
	Delt sin^a^	40	++	+++	+++	8%	+	989	3.23
	Delt sin	45	+++	+++	nd	6%	++		
EO3	Vastus sin	46	+++	nd	nd	nd	+		
	Delt^a^	58	+	++	++	0.5%	−	5703	4.74
	Delt sin^a^	60	+	++	++	2%	−	8311	7.22
EO4	Biceps sin	39	+++	+	+	6%	+	1393	0.699
	Vastus sin^a^	42	+++	+++	+++	1%	++	826	1.04
EO5	nd	48	+++	+	++	3%	+	1652	1.67
	Tib ant sin	49	+++	−	+	0.5%	+	1402	0.129
	Vastus dx^a^	53	+++	++	++	5%	+	2067	1.19
	Tib ant dx^a,b^	53	++	+	+	0.5%	+		
	Vastus dx^a,b^	53	++	++	++	10%	+	1355	6.20
	Biceps sin^a,b^	53	++	++	+++	2%	+	1435	1.91
EO6	Tib ant sin^a^	44	+	+++	+++	8%	+++		
	Delt sin	45	++	++	++	5%	−	5591	7.84

Inflammation (endomysial inflammatory cell infiltration): +, slight = 1 infiltrate/10 mm^2^; ++, moderate = 2–5 infiltrates/10 mm^2^; +++, intense ≥ 5 infiltrates/10mm^2^. RV: fibers with rimmed vacuoles: −, none; + , occasional; ++, several; +++, many. p62: Fibers with inclusions typical for IBM: −, not identified; + , occasional; ++, several; +++, numerous. COX deficiency: cytochrome c oxidase deficient fibers: + , few; ++, numerous; +++, large proportion (> 10%). Endomysial fibrosis: −, none; +, slight; ++, moderate; +++, marked. Copy number: mitochondrial DNA copy number in relation to nuclear DNA copy number. Mutation load: total sum of deletions and duplications in mitochondrial DNA

*EO* early-onset IBM, *y* years, *nd* not determined, *Delt* deltoid muscle, *Vastus* quadriceps muscle, vastus lateralis, *Tib ant* tibialis anterior muscle, *sin*, left, *dx* right

^a^Biopsy performed during immunomodulating treatment including corticosteroids

^b^Biopsies performed at the same day

### Mitochondrial DNA

**Fig. 3:**
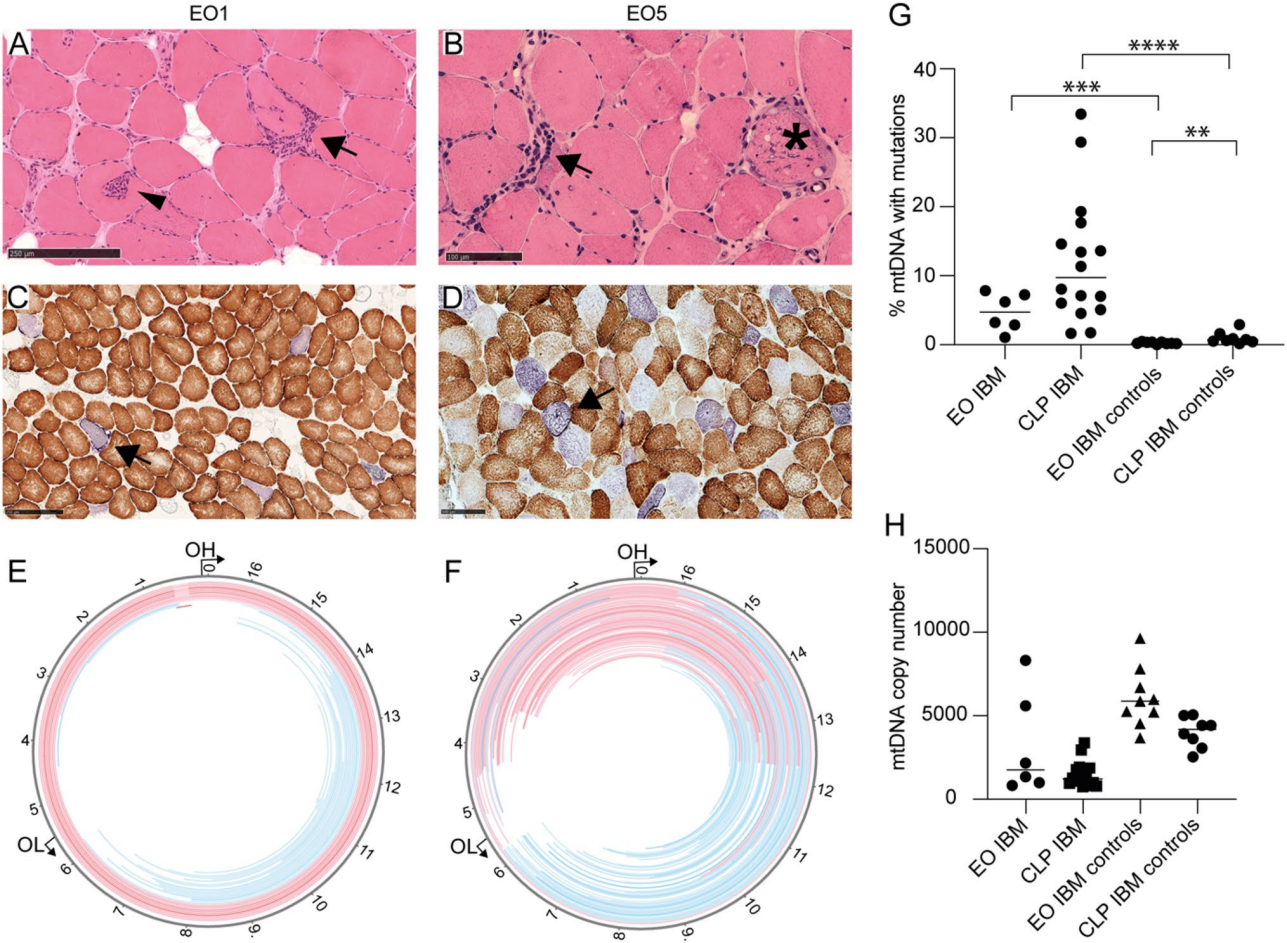
Morphological and mtDNA changes in early-onset IBM. **A**, **B** Hematoxylin and eosin staining shows inflammatory cell infiltrates (arrows), invasion of non-necrotic muscle fibers (arrowhead) and a fiber with rimmed vacuoles (asterisk). **C**, **D** There are numerous COX-deficient fibers, which appear blue (arrows) in cytochrome c oxidase/succinate dehydrogenase (COX/SDH) staining. **E**, **F** Circular map of muscular mtDNA in early-onset IBM with location of duplications (red) and deletions (blue). Higher color intensity relates to higher frequency of each rearrangement. Multiple rearrangements with different locations are present in the patients. Most deletions are located to the major arc between OH (origin of heavy strand replication) and OL (origin of light strand replication). **A**, **C**, **E** Muscle biopsy from the vastus lateralis muscle of patient EO1 at age 41 years, scale bar 250 μm. **B**, **D**, **F** Muscle biopsy from the vastus lateralis muscle of patient EO5 at age 53 years, scale bar 100 μm. **G**, **H** Mitochondrial DNA mutation load and mtDNA copy number in early-onset IBM, clinicopathological IBM and normal controls. The horizontal lines represent the median values. *EO* early-onset IBM, *CLP* clinicopathological IBM. **p = 0.0079, ***p = 0.0004, ****p < 0.0001

A total of 12 skeletal muscle biopsies from the six patients with early-onset IBM were available for analysis of mtDNA by deep sequencing, between one and five biopsies in each patient (Table 2). Median depth of coverage in patients and controls was 51,300× (range 11,111–229,530×). In patients with early-onset IBM, all analyzed samples showed multiple large-scale mtDNA deletions and duplications, corresponding to a total mtDNA mutation load ranging from 0.13 to 7.9%. The mtDNA copy number varied from 826 to 8311 (Fig. 3). No pathogenic single-nucleotide variants or small indels were found in the mtDNA.

The mtDNA mutation load level varied among different muscle biopsies also in the same individual with no clear correlation to time from symptom onset (Table 2). For each patient with early-onset IBM, the biopsy with the highest mutation load for large-scale rearrangements was used for comparison to avoid overestimation of the difference to clinicopathological IBM (Fig. 3). The median mutation load was much higher among patients with early-onset IBM and clinicopathological IBM compared to the corresponding controls. Although mtDNA copy number appeared considerably reduced in early-onset IBM compared to normal controls, no statistical analysis was performed due to the possible influence of inflammatory cell infiltrates on the ratio of nuclear DNA to mtDNA.

### Nuclear gene variants

Most variants identified after filtering were predicted to be benign or with uncertain significance according to ACMG Guidelines [14]. After manual assessment based on clinical information and muscle biopsy findings all variants could, with a high certainty, be excluded as disease causing in the patients.

Thirteen nuclear genes (*SPG7, DNA2, RNASEH1, SLC25A4, OPA1, TWNK, POLG, POLG2, TYMP, RRM2B, TK2, MPV17* and *DGUOK*) known to be associated with multiple large-scale rearrangement in mtDNA were investigated in more detail [15]. No patients with early-onset IBM harbored any pathogenic or likely pathogenic variants in coding or intronic regions in these genes.

## Discussion

In this study, we describe survival, epidemiology, clinical characteristics and mitochondrial DNA alterations in a population-based group of six individuals with early-onset IBM, verified with both muscle biopsy and clinical findings and fulfilling the ENMC 2011 criteria for clinicopathologically defined IBM except for the age critera [1].

Using the same population, method and period of time, 128 patients were diagnosed with clinicopathological IBM and three patients with clinicopathological IBM with unknown age at onset [6].

Several studies published during the last years have shown decreased survival in patients with IBM [6, 16–18]. Cumulative survival of early-onset IBM initially seemed to be similar to the population, suggesting that patients with early-onset IBM may be less vulnerable than patients with IBM during the early stages of disease. However, after approximately 10 years of follow up from diagnosis, survival rapidly decreased in early-onset IBM and became similar to clinicopathological IBM (Fig. 1). Even though the survival from diagnosis is longer in early-onset IBM (mean and median both 14 years) than in clinicopathological IBM (mean 9 years), the low median age at diagnosis (43 years in early-onset IBM compared to 79 years in clinicopathological IBM) results in a younger age at death for patients with early-onset IBM. The median age at death was only 59 years in early-onset IBM. This further emphasize the severe nature of the disease.

Both prevalence and incidence of early-onset IBM were considerably lower than for clinicopathological IBM. This finding is in line with the concept that IBM is a disease primarily affecting middle-aged and older individuals and supports that age limits in diagnostic criteria for IBM, usually varying from > 30 to > 45 years [1, 19], include a vast majority of patients with IBM [6]. The true prevalence and incidence of early-onset IBM might be higher than described in this study. Three patients fulfilling the ENMC 2011 criteria for clinicopathologically defined IBM except the age criteria had a first biopsy showing endomysial inflammatory cell infiltration at 59, 62 and 75 years respectively. These three patients fell outside the inclusion criteria for this study that had been designed aiming to include only patients with early-onset IBM verified with muscle biopsy with inflammation < 50 years of age. Although all three were diagnosed with IBM, it is uncertain if early symptoms of muscle weakness and/or dysphagia were due to other causes before later development of IBM, or the start of a slowly progressive disease. If included, the prevalence was 2.4 patients per million inhabitants.

Dysphagia is a common symptom in IBM [6, 20, 21]. While frequent in elderly individuals in the general population [22], it is rare among young healthy individuals. Interestingly, dysphagia was reported in five of six patients with early-onset IBM. This finding supports dysphagia as an important symptom of IBM for all age groups.

Respiratory dysfunction, requiring ventilation assistance, was reported in three of the six patients (50%) with early-onset IBM, but only in 8% of patients with clinicopathological IBM in the same population [6]. Fewer comorbidities and a tendency to initiate ventilation assistance more often in young patients might affect these numbers. Longer survival from symptom onset might also cause a larger number of patients with early-onset IBM developing respiratory dysfunction compared to clinicopathological IBM [6].

The progression rate of muscular weakness in IBM has been described in 66 patients by Lindberg et al., partially overlapping with the cohort of patients with early-onset IBM, with a mean decrease in knee extension of 1.22 Newton or 1.12% per month [23]. We found that the mean decline in knee extension strength in early-onset IBM correlated to the time from diagnosis and there was a wide range in the progression rate between the individual patients. In weaker patients, small absolute changes in strength gave large percentual differences (Fig. 2). The progression rate of knee extension weakness seems to be similar in early-onset IBM and IBM but needs to be further studied.

Autoimmune diseases appear more common in IBM than the general population [6, 16, 17, 24], and this seems to be the case also in early-onset IBM. Using the definition from Eaton et al., one of six patients with early-onset IBM had an autoimmune disease, similar to 21% in the corresponding group of 128 patients with clinicopathological IBM and higher than the lifetime prevalence of 5.3 in the Danish general population [6, 7]. If Raynaud syndrome was included, 50% of the patients with early-onset IBM had an autoimmune disease. This finding supports the role of autoimmunity in IBM. The occurrence of myositis associated autoantibodies was not possible to relate to symptoms or signs due to the retrospective study design. Malignancy was only seen in one patient (a basal-cell carcinoma) and does not seem to be an important factor in the pathogenesis of early-onset IBM.

All patients with early-onset IBM had tried both steroids and one or more immunomodulating treatment. As expected, this is higher than reported in IBM, as younger patients generally have fewer contraindications and higher tolerance for treatment. The present study can therefore not be regarded as a true natural history study. However, no treatment has proven efficient on group-level in IBM, which is probably true also in early-onset IBM. The reported transient effect of treatment seen in five patients was not possible to analyze in detail due to the retrospective study design, and might reflect a true treatment effect, a placebo response to or a combination of the two.

Mitochondrial changes in IBM were first described by Carpenter et al. [25], and later found to be associated with multiple mtDNA deletions and possibly also to reduced mtDNA copy number [10, 26]. Since multiple mtDNA deletions also occur in normal ageing, it has been hypothesized that they may be a sign of accelerated ageing in IBM partly based on the theory that IBM is primarily a degenerative age-related myopathy [27].

In this study we showed that in early-onset IBM, very high mutation load of large scale mtDNA rearrangements occur. In one patient the mutation load was close to 8% of all mtDNA already at 45 years of age. This finding strongly indicates that it is the muscle disease itself that triggers the formation of mtDNA rearrangements, presumably during mtDNA replication. As in our previous studies on IBM [10, 28], we did not find pathogenic variants in genes encoding proteins involved in mtDNA replication and known to cause multiple mtDNA deletions when mutated in patients with early-onset IBM. Therefore, other mechanisms are involved, which may be related to inflammation [10].

COX deficient muscle fibers and large-scale mtDNA rearrangements were a consistent finding in our biopsies from patients with early-onset IBM, but to a variable extent in different muscle biopsy samples including biopsies from the same muscle, even at the same age. This finding indicates that there is regional variability. A clinical significance on muscle function of the mtDNA mutations in IBM is likely, since the mutation load in some patients with IBM overlaps with those found in myopathy due to single mtDNA deletions [29]. In addition, it has been demonstrated that the mitochondrial changes most likely contribute to muscle fiber atrophy [30].

The low number of patients with early-onset IBM is a limitation in this study. However, it is the first population-based description of patients with early-onset IBM and while the patients were few, they are a part of a larger cohort, enabling comparison between groups as well as epidemiological analysis. All patients had a typical disease course, distribution of weakness and histopathological pattern for IBM, but due to the unusually early onset, whole genome sequencing was performed in all patients. This analysis, on its own, does not completely rule other muscle diseases such as facioscapulohumeral muscular dystrophy and some repeat expansion myopathies, but the combined clinical and morphological studies made these highly unlikely. We are not aware of any other studies on IBM, including patients with early-onset, where myopathy-associated nuclear genes have been systematically taken into consideration. We believe this approach is important when discussing outliers in studies on IBM and other muscle diseases.

In conclusion, early-onset IBM is a severe inflammatory myopathy with a high mitochondrial DNA mutation load in muscle fibers, causing progressive muscle weakness and a reduced cumulative survival in young and middle-aged individuals.

## Data Availability

The data supporting the findings are available (anonymized) upon reasonable request.
